# A False Alarm Reduction Method for a Gas Sensor Based Electronic Nose

**DOI:** 10.3390/s17092089

**Published:** 2017-09-12

**Authors:** Mohammad Mizanur Rahman, Chalie Charoenlarpnopparut, Prapun Suksompong, Pisanu Toochinda, Attaphongse Taparugssanagorn

**Affiliations:** 1 School of Information Computer and Communication Technology, Sirindhorn International Institute of Technology, Thammasat University, Khlong Luang 12121, Thailand; chalie@siit.tu.ac.th (C.C.); prapun@siit.tu.ac.th (P.S.); 2 Electronics and Communication Engineering Discipline, Science Engineering and Technology School, Khulna University, Khulna 9208, Bangladesh; 3 School of Bio-chemical Engineering and Technology, Sirindhorn International Institute of Technology, Thammasat University, Khlong Luang 12121, Thailand; pisanu@siit.tu.ac.th; 4 ICT Department, Telecommunications, School of Engineering and Technology, Asian Institute of Technology, Pathum Thani 12120, Thailand

**Keywords:** electronic nose, false alarm, hyperspheric classification boundary, classification, correct rejection

## Abstract

Electronic noses (E-Noses) are becoming popular for food and fruit quality assessment due to their robustness and repeated usability without fatigue, unlike human experts. An E-Nose equipped with classification algorithms and having open ended classification boundaries such as the *k*-nearest neighbor (*k*-NN), support vector machine (SVM), and multilayer perceptron neural network (MLPNN), are found to suffer from false classification errors of irrelevant odor data. To reduce false classification and misclassification errors, and to improve correct rejection performance; algorithms with a hyperspheric boundary, such as a radial basis function neural network (RBFNN) and generalized regression neural network (GRNN) with a Gaussian activation function in the hidden layer should be used. The simulation results presented in this paper show that GRNN has more correct classification efficiency and false alarm reduction capability compared to RBFNN. As the design of a GRNN and RBFNN is complex and expensive due to large numbers of neuron requirements, a simple hyperspheric classification method based on minimum, maximum, and mean (MMM) values of each class of the training dataset was presented. The MMM algorithm was simple and found to be fast and efficient in correctly classifying data of training classes, and correctly rejecting data of extraneous odors, and thereby reduced false alarms.

## 1. Introduction

Trained human sniffers are employed as quality checkers in industries. However, an expert nose can fatigue due to sickness or exposure to an odor for long time. To overcome this prostration of human beings, an electronic nose (E-Nose) was introduced in 1982 [1]. E-Noses can also be deployed in chemical areas unreachable by humans or animals. An E-Nose performs its operation unceasingly through its lifetime without fatigue. The essential constituents of an E-Nose are a sensing system (combination of a sensor panel and a data acquisition system) and an automated pattern classification system [2]. An efficient, fast, and simple classification algorithm is required for training an E-Nose, which classifies types of target objects with an acceptable error rate.

Data from any trained odor class should be correctly classified, and data from any irrelevant odor class should be correctly rejected. Any incorrect classification of the data from trained classes or irrelevant classes results in classification error. E-Nose classification errors can be categorized into two following kinds:

(i) Misclassification or false negative: odor data of a training class is classified to another trained or irrelevant class, and,
(ii) False classification or false positive: odor data of an irrelevant class classified to a training class.

Any false classification causes false alarm. Along with classification efficiency (correct classification rate), and speed of classification and simplicity, false classification rate and correct rejection rate should also be considered as criteria to choose a classification algorithm for E-Nose applications. Until the recently, few studies addressed “false classification” or “false alarm” in E-Noses. The false alarm issue has been addressed for fire detection in [3,4,5]. In [3,4,5] multilayer perceptron neural network (MLPNN), principal component analysis (PCA), and linear discriminant analysis are used for classification, respectively. Chemical agent detection and concentration estimation has been studied in [6], where authors have used a threshold based approach for false alarm detection, not considering the presence of irrelevant gases or VOCs. The sensors used for E-Nose applications are sensitive to a wide range of gases or VOCs. A false alarm is generated by an E-Nose if it comes in contact with any irrelevant gases or VOCs to which the E-Nose sensors are sensitive. Therefore, once the odor data are converted to electronic signals and preprocessed, the pattern recognition and decision making stage of the E-Nose must play an effective role for correct classification of the odor, as well as false alarm reduction. A scrupulous pattern recognition method is essential for extracting meaningful information from the sensed data. In [7,8,9] PCA is used for dimensionality reduction and visualizing data in reduced diemnsions. Classification algorithms, such as *k*-nearest neighbor (*k*-NN) [10,11,12,13], support vector machine (SVM) [11,12,13,14,15,16], generalized regression neural network (GRNN) [17], radial basis function neural network (RBFNN) [18,19,20,21,22], and MLPNN [9,20,21,22,23,24], etc., have been applied for odor classification by different E-Noses. Among these algorithms, *k*-NN, SVM, and MLPNN separate the classes by unbound classification boundaries, i.e., lines, planes, and hyper-planes in two, three, and higher dimensional space, respectively. A hyperspheric classification algorithm presented by Cooper [25] classifies a test data to a class if it resides within a predefined Euclidean distance from the class center, i.e., mean point of the class; else it is decided to be out of the class. An inversion algorithm for MLPNN [26] and a Gaussian kernel based SVM [27] are shown to generate closed boundary around training data which classify two classes of data, i.e., one class inside the boundary and another outside the boundary. The classification boundaries produced by RBFNN and GRNN with Gaussian activation functions are bounded in two, three, or higher dimensions. For more than three dimensions, they produce a bounded hyperspheric classification boundary. It is shown in this paper that the classification methods with open ended classification boundaries falsely classify irrelevant data to training classes, which cause false alarms and thus are not a good choice for E-Nose applications. A classification algorithm with a hyperspheric classification boundary is a potential candidate for reducing false classification rates, and thereby improves “correct rejection” rate in E-Nose applications.

In this paper, the *k*-NN, SVM, GRNN, RBFNN, and MLPNN were compared in terms of computational speed, false classification rate, correct classification rate, and correct rejection rate. It was observed that RBFNN and GRNN are suitable to cope with false alarms, with GRNN performing better than RBFNN. A simple hyperspheric classification algorithm based on maximum, minimum, and means (MMM) of each feature or variable (sensor) for each class was also proposed. The design complexity of the proposed MMM method was lower compared to *k*-NN, SVM, GRNN, RBFNN, and MLPNN classification methods. In addition, the MMM method was found to potentially reduce false classification errors. For the analyses in this research, fruity odor data were collected from four kinds of fruits namely, banana, mango, sapodilla, and pineapple at three ripeness states. Ripeness and quality of different kinds of fruits are also studied in [28] to assess apple quality during shelf life, in [29] to classify ripeness states of peaches and pears, in [30,31,32] to assess peach quality, in [33] for disease detection of blueberry, in [34] to assess ripeness and damaged blueberries, in [35] to assess the ripeness state of pink lady apples, in [36] online discrimination among banana, wine and baseline response is presented, and in [37] a review on food and fruit process monitoring, shelf-life investigation, freshness evaluation, and authenticity assessment is presented.

An E-Nose design process, sample collection, sensor array, experimental setup, data acquisition, and classification algorithms are discussed in materials and methods section. The materials and methods section is followed by the results and discussion. Throughout this paper individual capital bold and small bold letters are used to represent matrices and vectors, respectively.

## 2. Materials and Methods

An E-Nose design process, sample collection, design of the sensor array, experimental setup, data acquisition, and classification algorithms are discussed in this section.

### 2.1. E-Nose Design Process

**Figure 1:**
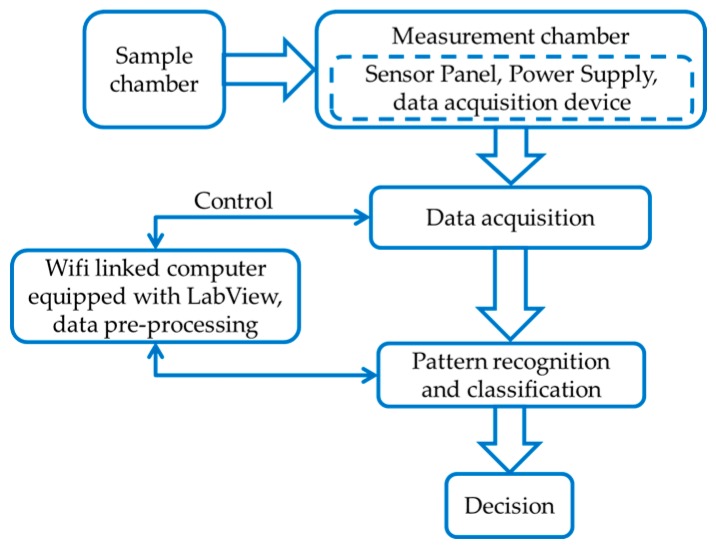
Design process of an electronic nose.

An E-Nose designing process has three main steps: (i) designing the sample and measurement chambers; (ii) data pre-processing; and (iii) classification and decision mapping. A block diagram of the design process is shown in Figure 1. The object under test was kept into the sample chamber, and the sensor array and data acquisition device were placed into measurement chamber. LabVIEW, and data processing and classification algorithms were installed in a Wi-Fi-connected computer to control the acquisition device, store data, pre-process the stored data to prepare signature patterns for training a classification algorithm, and classify odor data. The computer contained an Intel core i5 processor, 4 Gb random access memory, and ran with a 64 bit Windows 7 operating system. The outputs from the classification algorithm were mapped to the corresponding decisions set by the designer.

### 2.2. Sample Collection

**Figure 2:**
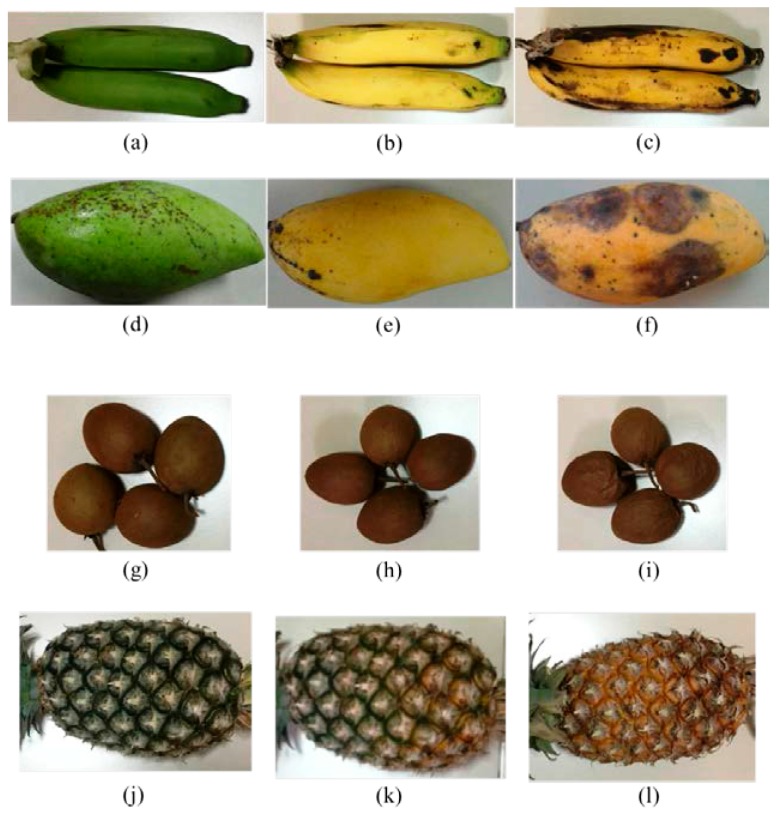
Fruit samples at different ripeness states: (**a**) unripe banana; (**b**) ripe banana; (**c**) rotten banana; (**d**) unripe mango; (**e**) ripe mango; (**f**) rotten mango; (**g**) unripe sapodilla; (**h**) ripe sapodilla; (**i**) rotten sapodilla; (**j**) unripe pineapple; (**k**) ripe pineapple; and (**l**) rotten pineapple.

Four categories of ripe fruits namely, banana, mango, sapodilla, and pineapple were chosen for this research. The photos of the fruits collected during experiment at their three ripeness stages are shown in Figure 2. Each fruit type was kept into the sample chamber in turn during the experiment. The samples were stored in different air tight boxes to prevent odor mix up. Throughout the experiment the fruits were preserved at 28 °C under normal conditions.

### 2.3. Sensor Panel

**Figure 3:**
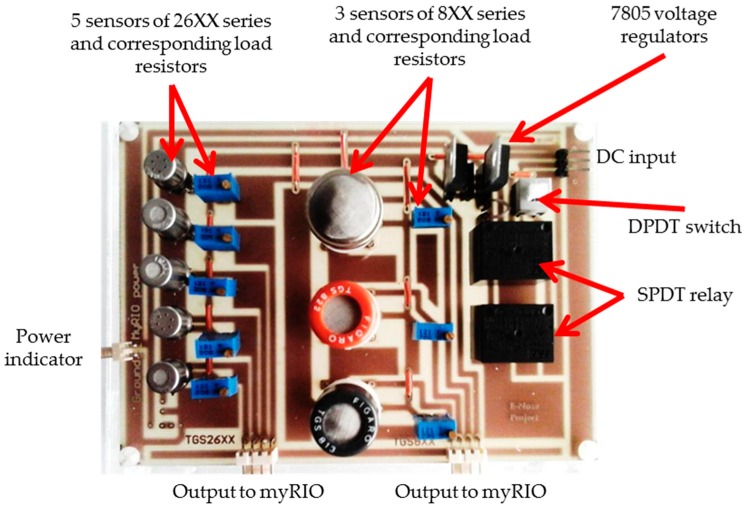
The electronic nose sensor panel. In the figure DC, DPDT, and SPDT stands for direct current, double pole double throw, and single pole double throw, respectively.

Eight metal oxide gas (MOG) sensors from FIGARO Engineering Inc. (Osaka, Japan), namely, TGS2612, TGS821, TGS822, TGS813, TGS2602, TGS2603, TGS2620, and TGS2610 were combined to build the sensor panel. The sensitivity of the sensors to different VOCs are summarized in Table 1, and the printed circuit board of the E-Nose sensor panel is shown in Figure 3. In Table 1, S1 to S8 are labels assigned to the sensors in this research. The sensors in the panel were altogether sensitive to ethylene, acetylene, propane, butane, hydrogen, hydrogen sulfide, carbon monoxide, benzene, ammonia, acetone, hexane, trimethyl amine, and methyl mercaptan. The sensors’ resistivity increased in the presence of free air or oxygen, and decreased in presence of target VOCs or gases. In presence of VOCs or gases, a current travelled through a sensor as in Figure 3, and the corresponding load resistor increased. As a result, the voltage across the load resistor of a sensor increased, corresponding to the concentration levels of VOCs or gases.

### 2.4. Experimental Setup

**Figure 4:**
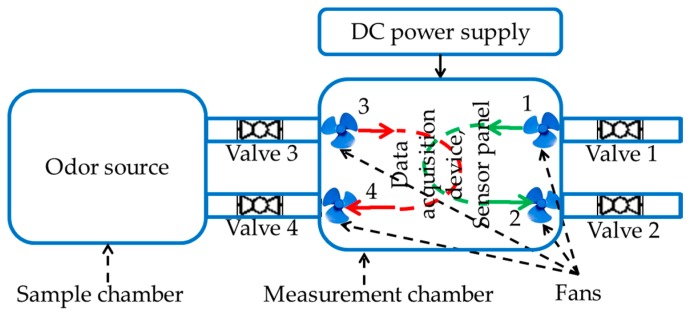
The E-Nose experimental setup. Valves 1 and 2, and Fans 1 and 2 are for air flow control, Valves 3 and 4, and Fans 3 and 4 are for circulating the odor between sample chamber and measurement chamber. A DC power supply powers the sensors, fans, and the data acquisition device. In the figure DC stands for direct current.

The fruit samples were kept in turn in the sample chamber shown in Figure 4. The sensor panel and the data acquisition device were kept into the measurement chamber. A LabVIEW installed personal computer (PC) was is wirelessly connected to the acquisition device to collect and pre-process the data. To prevent the mixing of undesired odors, both the chambers were air tightened. Connecting cables from a 5 volt and a 10 volt external direct current (DC) power supply are inserted through an airtight hole into the measurement chamber to power the sensors and the data acquisition device. The measurement process is comprised of two different phases, i.e., concentration and measurement. During concentration phase, Valves 3 and 4 were closed and VOCs were concentrated in the sample chamber. The Valves 1 and 2 were opened and Fans 1 and 2 were switched on during the concentration phase to clean the measurement chamber with free air and thereby neutralize the sensors. During the measurement phase, Valves 1 and 2 were closed, Valves 3 and 4 were opened, and Fans 3 and 4 were turned on to circulate odor VOCs between the samples and measurement chambers.

### 2.5. Data Acquisition

A data acquisition device from National Instruments Inc., named myRIO, was employed for data acquisition in the experiment. The acquisition device was controlled by a LabVIEW equipped PC via a wireless access point. Twenty repeated experiments were performed for each kind of fruits at three ripeness states, i.e., unripe, ripe, and rotten, which gave 240 measurements in total. The sensors were preheated for five minutes before beginning the experiment. The experiments were conducted in the following sequence: VOCs were concentrated for three minutes in the sample chamber, followed by two minutes of sensing. During the concentration phase, the measurement chamber was cleaned with free air to achieve sensor base level responses. To collect each data sample, this experiment cycle was repeated. At each ripeness state of the fruits, distinct transient and steady state responses were observed. Time domain sensor responses of an experiment to rotten sapodilla are given in Figure 4. The steady state responses of the sensors were used to produce the signature patterns for each fruit type at each ripeness state.

### 2.6. Pattern Recognition and Classification Algorithms

Classification algorithms as applied to E-Nose, namely, principal component analysis (PCA), *k*-NN, SVM, GRNN, RBFNN, MLPNN, and the proposed MMM method are presented in this subsection. The PCA is an unsupervised method of classification. In this research, supervised models were used for *k*-NN, SVM, GRNN, RBFNN, MLPNN, and MMM. The data matrix **X** and the corresponding target vector **t** (whose elements are class labels) are expressed as in Equations (1) and (2):

(1) $$X=\left[\begin{array}{c} X_{1}\\ X_{2}\\ \vdots \\ X_{m}\\ \vdots \\ X_{M} \end{array}\right]\;\text{and}\;t=\left[\begin{array}{c} t_{1}\\ t_{2}\\ \vdots \\ t_{m}\\ \vdots \\ t_{M} \end{array}\right],$$

where

(2) $$X_{m}=\left[\begin{array}{ccccc} x_{1,m,1} & \cdots & x_{1,m,n} & \cdots & x_{1,m,N}\\ x_{2,m,1} & \cdots & x_{2,m,n} & \cdots & x_{2,m,N}\\ \vdots & \cdots & \vdots & \cdots & \vdots \\ x_{l,m,1} & \cdots & x_{l,m,n} & \cdots & x_{l,m,N}\\ \vdots & \cdots & \vdots & \cdots & \vdots \\ x_{L,m,1} & \cdots & x_{L,m,n} & \cdots & x_{L,m,N} \end{array}\right]\;\text{and}\;t_{m}=\left[\begin{array}{c} t_{1,m}\\ t_{2,m}\\ \vdots \\ t_{l,m}\\ \vdots \\ t_{L,m} \end{array}\right],$$

*l* = 1, 2, …, *L* are the experimental indices of each class *m* = 1, 2, …, *M*; *L* is the number of data samples in each class, *M* is the number of classes, *n* = 1, 2, …, *N* is the sensor index, and *N* is the number of sensors. The rows in **X** are experimental samples and the columns are feature or sensor variables. The training, validation and testing data are chosen from **X** as per the ratio defined by the designer. From each class, 70%, i.e., 0.7*L* samples are taken for training the algorithms.

#### 2.6.1. Principal Component Analysis

PCA [4,7,8,9,10,11] is an unsupervised method of data analysis which reduces dimensionality while preserving maximum data variance. PCA converts the data to a new feature space through an orthogonal transformation process. The direction of maximum data variance in the original space is the first principal component (PC 1), the direction of second largest data variance which is orthogonal to the PC 1 is the second principal component (PC 2), PC 3 is orthogonal to both PC 1 and PC 2, and so on for higher dimensional PCs. The algorithm is given as follows:

**Step** **1.** Find the mean vector **x** = [*x*_1_, *x*_2_, …, *x_N_*], where each element represents the mean of each column of **X**. *N* is the number of sensors.
**Step** **2.** Deduct the mean vector *x* from each row of matrix **X**.
**Step** **3.** Find the covariance matrix of the mean deducted matrix in Step 2.
**Step** **4.** Find the eigenvalues and eigenvectors of the covariance matrix found in Step 3.
**Step** **5.** Sort the eigenvectors in descending order of eigenvalues.
**Step** **6.** The eigenvector with maximum eigenvalue is PC 1, the eigenvector corresponding to the second largest eigenvalue is PC 2, and so on for the descending eigenvalues.

PCA was used in this paper for visualizing higher dimensional data by reducing dimensionality.

#### 2.6.2. *k*-Nearest Neighbor 

*k*-NN [10,11,12,13] is the simplest pattern recognition and classification algorithm of all the machine learning algorithms. During the training phase a fraction, i.e., 70 percent of the experimental samples from matrix **X** in Equation (3) are chosen for training. The feature vectors in matrix **X** and the class labels in the target vector **t** in Equation (3) corresponding to training data, are stored during the training phase. The remaining 30 percent of the dataset is used for testing. The nearest *k* training samples from the test data vector are found first. An Euclidean distance metric is used to measure the closeness. The test data is assigned to the winning class by a majority voting between the closest *k* neighbors. To avoid any possible tie, *k* must be an odd number, and *k* must not be a multiple of the number of classes. The computational complexity of *k*-NN is *O*(*LM*(*N* + *k*)). A supervised *k*-NN model is used in this research.

#### 2.6.3. Support Vector Machine

For the SVM [11,12,13,14,15,16] classification, the hyperplanes that provide the maximum margin between two classes, having no interior data, were found. The data that lie on the hyperplanes are known as support vectors. A hyperplane which lies between these two hyperplanes, so that the distances from it to the support vectors of both the classes are maximized, is known as a maximum-margin hyperplane. A linear classifier defined by the maximum-margin hyperplane is known as a maximum margin classifier. The SVM algorithm is given as follows:

**Step** **1.** Maximize the Lagrangian [Equation (3)] such that $\sum_{m}\sum_{l}\alpha_{l,m}t_{l,m}\geq 0$ and $\alpha_{l,m}\geq 0$.
  (3) $$L\left(\alpha_{l,m}\right)=\sum_{l,m}\alpha_{l,m}-\frac{1}{2}\sum_{l,m,g,j}\alpha_{l,m}\alpha_{g,j}t_{l,m}t_{g,j}\left(x_{l,m}\times {\;x}_{g,j}\right),$$
  where *l* and *g* are experiment indices within a class, *m* and *j* are class indices, *α_l_*_,*m*_ are Lagrange multipliers, *t_l_*_,*m*_ are class labels (+1 or −1), and **x** are data vectors.
**Step** **2.** Find the value of *α_l_*_,*m*_ from Equation (3).
**Step** **3.** Put the values of *α_l_*_,*m*_ in Equation (5) to find the weight vector by Equation (4).
  (4) $$w=\sum_{l,m}\alpha_{l,m}t_{l,m}x_{l,m}$$
**Step** **4.** By the Karush–Kuhn–Tucker condition [38] as shown in Equation 5 below and weights found from Equation (6), bias *b* is calculated.
  (5) $$\alpha_{l,m}\left[t_{l,m}\left(w\times x_{l,m}+b\right)-1\right]=0,$$
**Step** **5.** Classify test data by looking at the sign of the solution to Equation (6) as given below:
  (6) $$f\left(u\right)=w\cdot u+b=\left(\sum_{s=1}^{S}\alpha_{s}t_{s}x_{s}\times u\right)+b,$$
  where, *s* is support vector index, *S* is the number of support vectors, *b* is the bias, and *u* is the data vector under testing.

The basic SVM is a binary classifier. In this research, multiple linear binary SVM classifiers were combined to obtain a multiclass SVM classifier. A multiclass SVM classifier classifies data with a majority voting between the outcomes from the binary SVM classifiers by the *k*-NN method.

The computational cost of solving the SVM problem has both a quadratic and cubic component. Based on small margin or large margin violations, SVM complexity varies between *O*(*NL*^2^*M*^2^) and *O*(*NL*^3^*M*^3^).

#### 2.6.4. Multilayer Perceptron Neural Network

**Figure 5:**
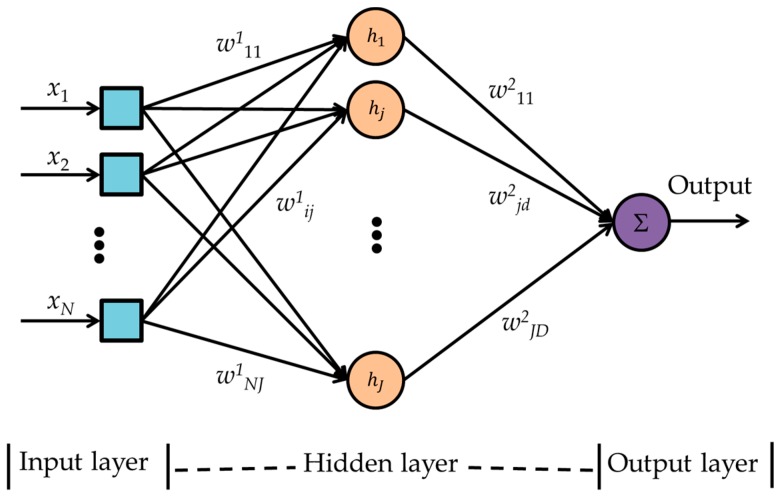
Multilayer perceptron neural network block diagram. *i* is the sensor index, *j* is the index for hidden layer neuron, and *d* is the index of output layer neuron.

The MLPNN [9,20,21,22,23,24] is a supervised feed-forward neural network that uses back-propagation training and generalized delta rule learning. Each layer of an MLPNN is fully connected to the next layer as a directed graph. After every epoch, the mean squared errors (MSEs) are calculated from the differences between the outputs of the validation dataset to the corresponding targets. To minimize the error in every epoch the synaptic weights and activation thresholds are adjusted. A three layer MLPNN as shown in Figure 5 is applied for this research. The input layer has eight (*N*) neurons corresponding to eight sensors. The hidden layer consists of 10 (*J*) neurons with tan-sigmoid (*h*) activation function. The output layer has one (*D*) neuron with linear activation function to provide class decision corresponding to an input data. The MLPNN training algorithm is given below.

**Step** **1.** Initialize the weights to small positive and negative random values.
**Step** **2.** Run the network forward with each training data to get the network outputs as given in Equation (7), and calculate the errors as shown in Step 3 to Step 6.
  (7) $$y_{l,m}^{3}=g\left(\sum_{j=1}^{J}w_{jd}^{2}h_{j}\left(\sum_{i=1}^{N}w_{ij}^{1}x_{l,m,i}\right)\right),$$
  where $h_{j}(a)=2/(1+\exp(-2a))-1$, *i* is the sensor index, *j* is the index for the hidden layer neuron, and *d* is the index of the output layer neuron.
**Step** **3.** Compute the backpropagation error terms for the links to the output neuron as in Equation (8) below:
  (8) $$\delta_{l,m}^{2}=y_{l,m}^{3}\left(1-y_{l,m}^{3}\right)\;\left(y_{l,m}^{3}-t_{l,m}\right).$$
**Step** **4.** For each hidden node, calculate the backpropagation error term as in Equation (9) below,
  (9) $$\delta_{j}^{1}=y_{j}^{2}\left(1-y_{j}^{2}\right)\sum_{d=1}^{D}\delta_{l,m}^{2}w_{jd}^{2}.$$
**Step** **5.** Update the synaptic weights from a node in Layer 1 to a node (neuron) in Layer 2, as Δ*w*^1^*_ij_* = −*ηδ*^1^*_j_x_l,m,n_*, and apply, *w*^1^*_ij_* = *w*^1^*_ij_ +* Δ*w*^1^*_ij_*. Update the synaptic weights from a node at Layer 2 to a node in Layer 3, as Δ*w*^2^*_jd_* = −*ηδ*^2^*_d_y_j_*, and apply, *w*^2^*_jd_* = *w*^2^*_jd_ +* Δ*w*^2^*_jd_*.
**Step** **6.** Calculate MSE by Equation (10) as follows:
  (10) $$MSE=\frac{1}{N_{ts}}\sum_{m=1,\;l=1}^{M,\;0.15L}{\left(y_{m,l}^{3}-t_{m,l}\right)}^{2},$$
  where *N*_ts_ is total number of test samples, and *L* is multiplied by 0.15, as 15% of the data from each class in matrix X are utilized for validation.
**Step** **7.** Repeat from Step 2 until the error limit or the number of the epoch limit is reached.

After training and validation, 15% remaining experimental data samples are passed through the network to test the network classification performance.

Assumptions:

- w^1^*_ij_* are weights from node *i* of layer 1 (input layer) to node *j* of layer 2 (hidden layer),
- w^2^*_jd_* are weights from node *j* of layer 2 to node d of layer 3 (output layer),
- hidden layer neurons’ activation functions are sigmoid transfer functions,
- y^2^*_j_* is the output from node *j* of layer 2,
- y^3^*_l,m_* is the output from the output layer,
- *t_l,m_* is the corresponding target,
- *η* is the learning rate,

Biases to any neuron in Layer 2 are considered to be 1 (not shown in the algorithm and in Figure 5 for simplicity.) The computational cost of MLPNN is *O*(*I*^2^_MLPNN_)where *I*_MLPNN_ is the total number of neurons in the MLPNN.

#### 2.6.5. Generalized Regression Neural Network

**Figure 6:**
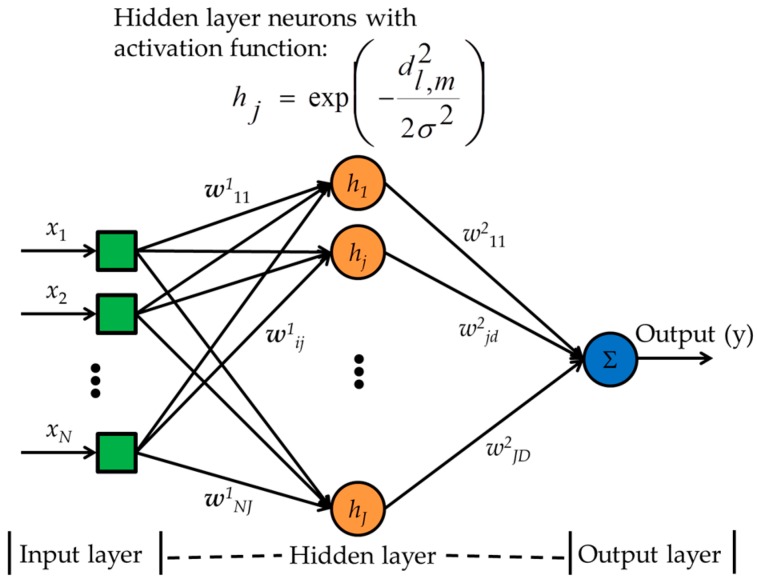
A generalized regression neural network block diagram. *d*^2^*_l_*_,*m*_ is Euclidean distance and *σ* is the spreading factor.

A GRNN [17], a supervised neural network model, has three main layers namely, input, hidden, and output layers, as shown in Figure 6. At the beginning of the training phase, the synaptic weights (w^1^*_ij_*) from the input to the hidden layer neurons are initialized to the training data vectors and the synaptic weights (w^2^*_jd_*) from the hidden layer neurons to the output neurons are initialized to the targets corresponding to the training data. An input is fed by the input layer to the neurons in the hidden layer also known as pattern layer or radial basis layer. At the hidden layer, the Euclidean distances (weights) from the training data to the test data are calculated and processed by a Gaussian activation function to produce hidden layer outputs. A larger Euclidean distance results in smaller weights, and a smaller distance results in larger weights. At the output layer, the weighted summation of the hidden layer outputs (normalized by the summation of the hidden layer outputs) are calculated. This normalized output is passed through the linear activation function and the final output, i.e., the class of the test data, is achieved.

The GRNN training algorithm is given as follows:

**Step** **1.** Separate all the experimental samples in matrix **X** into 70% training samples, 15% validation samples, and 15% test samples.
**Step** **2.** Initialize the synaptic weights from the input nodes to the hidden layer neurons to the training samples, and the weights from the hidden layer neurons to the output neuron with the corresponding training targets in vector **t**.
**Step** **3.** Find the outputs to validation inputs **x***_v_*_,*m*_ by Equation (11) as:
  (11) $$y\left(x_{v,m}\right)=\frac{\sum_{l,m}t_{l,m}\exp\left(-\frac{d_{l,m}^{2}}{2\sigma^{2}}\right)}{\sum_{l,m}\exp\left(-\frac{d_{l,m}^{2}}{2\sigma^{2}}\right)},$$
  where **v** = 1, 2, …, 0.15*L*, is the validation data index in class *m*, *σ* is the spreading factor, and *d*^2^*_l_*,*_m_* = (**x***_v_*_,*m*_ –x*_l_*_,*m*_)^T^(**x***_v_*,*_m_* − **x***_l_*_,*m*_), [(.)^T^ indicates transpose] is the square Euclidean distance from a training sample **x***_i_*_,*m*_ to a validation sample **x***_v_*,*_m_*.
**Step** **4.** Calculate the MSE as:
  (12) $$E=\sum_{v,m}{\left(y\left(x_{v,m}\right)-t_{v,m}\right)}^{2}.$$
**Step** **5.** If *E* > *E_threshold_*, then adjust *σ*, and continue to Step 3, otherwise stop.

The computational cost of GRNN is *O*(*I*_GRNN_) where *I*_GRNN_ is the total number of neurons in the GRNN.

#### 2.6.6. Radial Basis Function Neural Network

The design of a supervised RBFNN [18,19,20,21,22] is similar to the GRNN. However, the weights from the hidden layer neurons to the output neuron, **w**^2^, are not the true targets, but are initialized to small random numbers when the training begins, and are updated to optimal values during the training phase to reduce output error. The RBFNN algorithm is presented below.

**Step** **1.** Choose a value for σ ranging randomly from 0 to 1.
**Step** **2.** Choose random weights for the weights from the hidden layer neurons to the output neuron, i.e., w2.
**Step** **3.** Calculate the entries of the Φ matrix, where Φ is defined as below:
  (13) $$\Phi =\left[\begin{array}{ccc} \exp\left(-\frac{{\left\|x_{1,1}-x_{1,1}\right\|}^{2}}{2\sigma^{2}}\right) & \cdots & \exp\left(-\frac{{\left\|x_{1,1}-x_{L,M}\right\|}^{2}}{2\sigma^{2}}\right)\\ \vdots & \ddots & \vdots \\ \exp\left(-\frac{{\left\|x_{L,M}-x_{1,1}\right\|}^{2}}{2\sigma^{2}}\right) & \cdots & \exp\left(-\frac{{\left\|x_{L,M}-x_{L,M}\right\|}^{2}}{2\sigma^{2}}\right) \end{array}\right],$$
  where, **x** are *N* dimensional vectors.
**Step** **4.** Calculate the weight vector, w2 = Φ − 1t.
**Step** **5.** Calculate output, y = w2Φ.
**Step** **6.** Evaluate, mean squared error, $E=\sum_{v,m}{\left(y\left(x_{v,m}\right)-t_{v,m}\right)}^{2}$
**Step** **7.** If E > Ethreshold, change σ and continue to step 4 to adjust w2, otherwise exit.

The computational cost of RBFNN is *O*(*I*_RBFNN_) if optimal weights are found in one epoch. If the number of epochs are high, the complexity can be up to *O*(*I*^2^_RBFNN_), where *I*_RBFNN_ is the total number of neurons in the RBFNN and is equal to *I*_GRNN_.

#### 2.6.7. Minimum-Maximum-Mean Hyperspheric Classification Method

The MMM method, a simplified hyperspheric classification method proposed in this paper for the E-Nose, is based on maximum, minimum, and mean responses of the sensors for different classes. This proposed MMM classification method is a supervised model. During training, the maximum vectors (${\vec{q}}_{C_{n}}$), minimum vectors (${\vec{v}}_{C_{n}}$), and mean vectors (${\vec{u}}_{C_{n}}$) are calculated from each class from the training data samples, and stored in matrices **Q**, **V**, and **U**, respectively as:

(14) $$Q={\left[\begin{array}{c} {\vec{q}}_{1}\\ \begin{array}{l} \vdots \\ {\vec{q}}_{C_{n}}\\ \vdots \end{array}\\ {\vec{q}}_{C_{N}} \end{array}\right]}_{C_{N}\times 1}={\left[\begin{array}{ccc} q_{C_{1},S_{1}} & \ldots & q_{C_{1},S_{N}}\\ \vdots & \ddots & \vdots \\ q_{C_{N},S_{1}} & \cdots & q_{C_{N},S_{N}} \end{array}\right]}_{C_{N}\times S_{N}},$$

(15) $$V={\left[\begin{array}{c} {\vec{v}}_{1}\\ \begin{array}{l} \vdots \\ {\vec{v}}_{C_{n}}\\ \vdots \end{array}\\ {\vec{v}}_{C_{N}} \end{array}\right]}_{C_{N}\times 1}={\left[\begin{array}{ccc} v_{C_{1},S_{1}} & \ldots & v_{C_{1},S_{N}}\\ \vdots & \ddots & \vdots \\ v_{C_{N},S_{1}} & \cdots & v_{C_{N},S_{N}} \end{array}\right]}_{C_{N}\times S_{N}},$$

(16) $$U={\left[\begin{array}{c} {\vec{u}}_{1}\\ \begin{array}{l} \vdots \\ {\vec{u}}_{C_{n}}\\ \vdots \end{array}\\ {\vec{u}}_{C_{N}} \end{array}\right]}_{C_{N}\times 1}={\left[\begin{array}{ccc} u_{C_{1},S_{1}} & \cdots & u_{C_{1},S_{N}}\\ \vdots & \ddots & \vdots \\ u_{C_{N},S_{1}} & \cdots & u_{C_{N},S_{N}} \end{array}\right]}_{C_{N}\times S_{N}}.$$

In Equations (14)–(16), *C_N_* and *S_N_* are the number of classes and number of sensors, respectively. The maximum, minimum, and mean vectors are the rows of the matrices **Q**, **V**, and **U**, respectively. The columns of the matrices **Q**, **V**, and **U** represent sensor variables. A test data vector is compared element by element, i.e., every feature is compared to the maximum and minimum vectors of each class. A test data is classified to a class for which the following two criteria satisfy: (i) every element (feature variable) of the test data vector is less than, or equal to every element of the maximum vector of the corresponding class, and (ii) every element of the test data vector is greater than, or equal to every element of the minimum vector of the corresponding class. The criteria (i) and (ii) ensure the minimum-maximum range assessment for each sensor by the logical ‘AND’ operation between all the sensor variables. The data are assigned to the classes for which the logical examinations are true. If any odor data is classified to multiple classes, the Euclidean distances between the test data and the class mean vectors of the corresponding tied classes in Matrix **U** are calculated. The test data are assigned to the class in which the mean vector is the closest to the test data vector. The algorithm is given as follows:

**Step** **1.** Compute the maximum, minimum, and mean matrices **Q**, **V**, and **U** from the whole dataset in **X**, where each row of the matrices **Q**, **V** and **U** corresponds to a class and each column corresponds to a sensor variable.
**Step** **2.** Compare each feature of a test data vector with each feature of the maximum and minimum vectors of all the classes.
**Step** **3.** Assign test data to a class for which the following two criteria satisfy: (i) each feature of the test data vector is less than or equal to the corresponding feature of the maximum vector of the corresponding class, and (ii) each feature of the test data vector is greater than or equal to each element of the minimum vector of the corresponding class. If the test data do not fall within the maximum-minimum range of any of the classes, then label it as “unclassified” or “correctly rejected”, and stop.
**Step** **4.** If any test data are within maximum-minimum limits of multiple classes, then the case of a tie occurs. To break this tie, the test data are assigned to the class whose mean vector (measured by Euclidean distance metric) is the closest to the test data vector. Once the test data are assigned to a class, exit the program.
**Step** **5.** Run the steps from 1 to 4 for the validation dataset and calculate the percentage of error by $E_{MMM}=\sum_{l=1,m=1}^{0.15L,0.15M}{\left(y_{l,m}-t_{l,m}\right)}^{2}$ If the error $E_{MMM}\leq E_{threshold}$ then exit, otherwise add $\eta \times \sigma_{m}$ to the corresponding rows of **Q** and deduct from *m*th row of **V** to expand the hyperspheric classification boundary and continue to Step 1, where, *η* is the learning rate, and *σ_m_* is the standard deviation vector of class *m*.

All sensors in the sensor array are required to function properly. A malfunctioning sensor should be detected and replaced, and the E-Nose should be trained again to achieve good classification performance. Each of the mean, minimum, and maximum matrices has a computational cost of *O*(*M*). If a tie occurs, the complexity becomes *O*(*M + I*_ties_(*N* + *k*)), where Ities is the number of tied classes. The latter terms are added as per *k*-NN theory to break the tie, and the class with closest mean is needed to be found by the *k*-NN method. Due to the E-Nose data pattern, ties are less likely to occur, and thereby the computational complexity remains low.

## 3. Results

**Figure 7:**
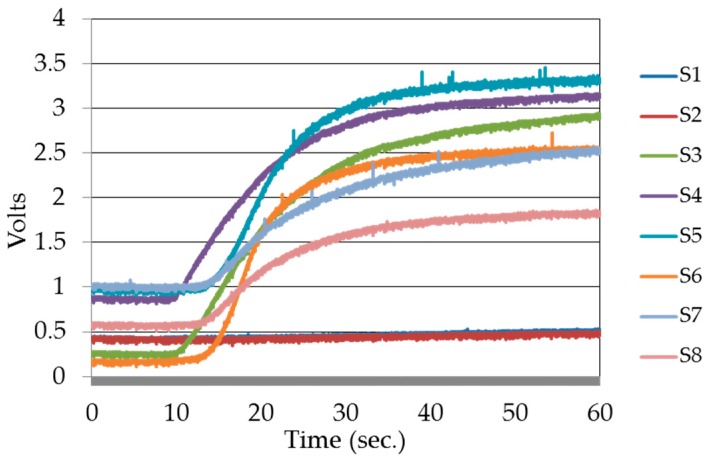
Time versus voltage responses of eight sensors to rotten sapodilla. S1 to S8 are sensor labels as listed in Table 1.

Electric responses, i.e., voltage changes across the sensor load resistors of the E-Nose sensor panel to the odors from four kinds of fruits, i.e., banana, mango, sapodilla, and pineapple, each at their three ripeness states forming twelve classes, were recorded for this research. One set of recorded time versus voltage response for ripe sapodilla is shown in Figure 7. Each set of response comprised responses from eight sensors. The data samples were prepared by averaging the steady state voltage responses from the sensors. The classification algorithms were trained with 70% of the data samples from banana, sapodilla, and pineapple, each at three different ripeness states (nine classes), and 30% of the data samples from each of the nine classes were used for validation and testing. Data samples from mango odor at three ripeness states were considered as three irrelevant classes, and were used to test false classification and correct rejection performances of the classification algorithms.

**Figure 8:**
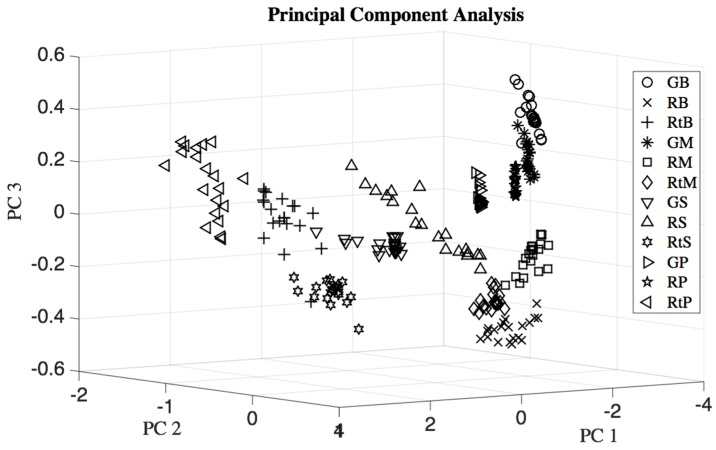
Three dimensional principal component analysis (PCA) scores plot of four types of fruits each at three ripeness states: GB = green banana, RB = ripe banana, RtB = rotten banana, GM = unripe mango, RM = ripe mango, RtM = rotten mango, GS = unripe sapodilla, RS = ripe sapodilla, RtS = rotten sapodilla, GP = unripe pineapple, RP = ripe pineapple, and RtP = rotten pineapple.

A three dimensional PCA scores plot of banana, mango, sapodilla, and pineapple at three ripeness states in Figure 8 shows that few samples of green banana and green mango, ripe mango and rotten mango, rotten banana and green sapodilla, rotten banana and rotten sapodilla overlapped with each other.

**Figure 9:**
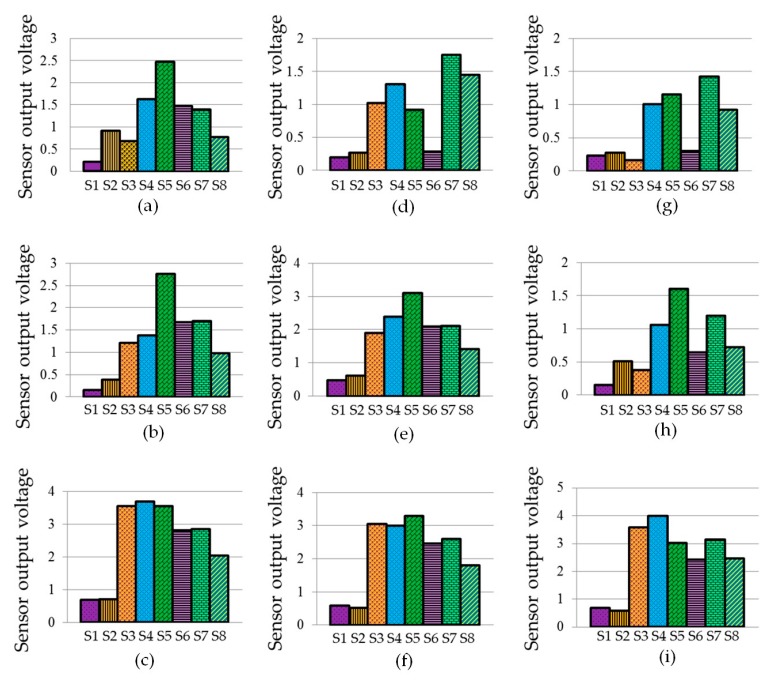
Signature patterns of the means of three types of fruits at three ripeness states: (**a**) green banana; (**b**) ripe banana; (**c**) rotten banana; (**d**) green sapodilla; (**e**) ripe sapodilla; (**f**) rotten sapodilla; (**g**) green pineapple; (**h**) ripe pineapple, and (**i**) rotten pineapple.

The signature patterns as depicted in Figure 9 were prepared by the mean responses of nine trained classes, i.e., green banana, ripe banana, rotten banana, green sapodilla, ripe sapodilla, rotten sapodilla, green pineapple, ripe pineapple, and rotten pineapple. The signatures were composed of eight features corresponding to the eight sensors. It was seen that the signatures were significantly different from each other. The responses of the sensors S1 and S2 were small, having insignificant variation for different classes. In contrast, the sensors S3 to S8 showed significant variations in responses for the different classes, and produced distinct signature patterns for different fruit odors and their ripeness states.

For training, validation, and testing, 70%, 15%, and 15% of the data samples were chosen from the trained classes in equal numbers, respectively, to train, validate, and test the MLPNN, RBFNN, GRNN, and MMM algorithms. Whereas, to train the *k*-NN and SVM algorithms, 70% of data samples were used for training, and 30% of data samples were used for testing as training; these algorithms did not need a validation step.

Training and testing time taken by different classification algorithms are shown in Table 2. The proposed MMM algorithm required minimum training time, followed by the *k*-NN, GRNN, SVM, MLPNN, and RBFNN algorithms, respectively. The testing time taken by the MMM classification method was also minimal, followed by the MLPNN, RBFNN, SVM, GRNN, and *k*-NN algorithms, respectively. Thus, training the proposed classification algorithm and classifying any test data was faster compared to the MLPNN, RBFNN, SVM, GRNN, and *k*-NN algorithms. The training and testing were faster with the MMM method, as less calculations were required to find the minimum, maximum, and mean vectors for each class, and the compression or expansion factor of the classification boundaries. The big ‘*O*’ complexities of the algorithms, according to Section 2.6, were *O*(*LM*(*N* + *k*)) for *k*-NN, within *O*(*NL*^2^*M*^2^) to *O*(*NL*^3^*M*^3^) for SVM, *O*(*I*^2^_MLPNN_) for MLPNN, *O*(*I*_GRNN_) for GRNN, within *O*(*I*_RBFNN_) to *O*(*I*^2^_RBFNN_) for RBFNN, and within *O*(*M*) to *O*(*M* + *I_ties_*(*N* + *k*) for the MMM method. This was the case because MLPNN and SVM both had square terms, which implied more computations and thereby more complexity. Although GRNN and RBFNN had low orders of complexity, as they need as many neurons as there were training data, the design complexity was also high. *k*-NN needed to be applied within the MMM method to break ties, and *I_ties_* were less likely to occur and were usually much smaller compared to *LM*, keeping the complexity of the MMM method small. Thus, the MMM method took less time compared to other algorithms analyzed in this research.

Test data from the trained classes are classified by the classification algorithms to compare their misclassification and correct classification performances are averaged over 10 simulations and summarized in Table 3. The proposed MMM, GRNN, *k*-NN, and SVM classification methods showed 1.8519% classification errors, while misclassification errors with the RBFNN and MLPNN methods were 24.0740% and 29.6296%, respectively.

In a one-way multiple analysis of variance (MANOVA) test, the maximum *p*-value of 1.1088 × 10^−10^ (which is very small compared to 5% significance level) occurred for eight dimensions. This indicates that the eight dimensional mean vectors of the twelve classes were obviously distinguishable from each other. This was the case because the MANOVA test assumes the variance-covariance matrix is the same for each population, which is not exactly true for real scenarios. Thus, the means were distinguishable, but the dataset had a few occurrences of minor overlap, as seen in the PCA plot in Figure 8 causing classification errors. In Table 3 it was seen that false negative errors occurred for different classification models.

The number of samples should not be too small compared to the number of features. In this research, the number of features was eight, and for each class, 20 samples were collected. To verify over-fitting of the classification models, the R-square statistics of the target outcomes from the models were evaluated for training, validation, and test datasets are listed in Table 4. The statistics showed that R-squared values were smaller for MLPNN and RBFNN classification models compared to *k*-NN, SVM, GRNN, and MMM classification models. These statistics validate the classification performances presented in Table 3.

False classification and correct rejection performances of the classification algorithms analyzed in this paper are summarized in Table 5. These analyses have not been evaluated in literatures yet, to the best of our knowledge. It was expected that the 60 samples, i.e., 100% odor data from the irrelevant (i.e., mango odor data at three ripeness states for this research) classes should have been correctly rejected by an E-Nose, and thereby would not produce false classification errors. The *k*-NN and SVM algorithms falsely classified all the data from irrelevant classes and produced false alarms. The RBFNN algorithm falsely classified 15% of the mango data to trained classes and misclassified 85% to unknown extraneous classes. The MLPNN algorithm falsely classified 35% of the data samples and misclassified 65% to unknown extraneous classes. It was seen that the proposed MMM method (with each feature value of each class in the maximum matrix increased by 11.11% of the corresponding class’s standard deviations) and the GRNN method (with a spreading factor of 0.03) do not show any false classification error, and all irrelevant data samples were correctly rejected.

## 4. Discussion

In this paper classification performances of the *k*-NN, SVM, MLPNN, RBFNN, GRNN, and the proposed MMM classification algorithms were compared in terms of computation speed, correct classification rate, and false classification rate. Correct classification performances experienced by different algorithms in this paper are shown in Table 3. These results were consistent with previous works, with true positive, i.e., correct classification accuracy ranges from 82.4% to 100% with *k*-NN [10,11,12,13], 86% to 98.66% with SVM [11,12,13,14,15,16], 100% with GRNN [17], 88% to 100% with RBFNN [18,19,20,21,22], and 68% to 100% with MLPNN [9,20,21,22,23,24]. With the MMM classification method, 98.1481% classification accuracy has been achieved.

Odor data from an irrelevant class should not be classified to any trained class by an E-Nose, and thereby produce no false alarm. False classification performances of the existing classification methods have not yet been analyzed in the literature. To reduce false classification and misclassification errors and improve correct rejection performance, classification algorithms with a hyperspheric boundary were used. It is seen from Table 5 that the hyperspheric classification algorithm, i.e., GRNN with Gaussian activation function, showed zero false classification errors. In contrast, RBFNN, another hyperspheric classification method with Gaussian activation functions in the hidden layer neurons, showed 15% false classification errors. Compared to this, the proposed MMM classification method was found to be efficient for E-Nose data classification. It is seen from the analysis as tabulated in Table 3 that the MMM method classified the test data of trained classes with only a 1.8519% misclassification error, i.e., false negative error. In addition, and similar to the GRNN, the MMM method correctly rejected data samples of irrelevant classes and thereby did not produce any false alarm. In addition, the implementation complexity, training, and testing duration of the MMM method was less compared to other methods analyzed in this paper. Thus, the MMM method could be a prominent method for E-Nose application, to improve classification rate of the data samples which belong to training classes, reduce false classification rates, and increase correct rejection rates of the data samples from irrelevant classes.

As the design of a GRNN is complex and expensive due to its high level of neuron requirement, a simple hyperspheric classification method based on minimum, maximum, and mean (MMM) values of the training dataset was proposed in this paper. It was seen that the MMM algorithm is simple, fast, and highly accurate for classifying data of trained classes and correctly rejecting data of extraneous odors. Although the MMM and GRNN methods showed similar misclassification and false classification performance, the MMM method is promising for E-Nose applications because of the simplicity and speed of its implementation.

## Figures and Tables

**Table 1:** Figaro gas sensors used in the E-Nose design and the gases or volatile organic compounds (VOCs) to which the sensors are sensitive.

Sensor Model	Sensor Label	Gases and VOCs											
		CH_2_	C_2_H_2_	C_3_H_8_	C_4_H_10_	H_2_	H_2_S	CO	C_6_H_6_	NH_3_	(CH_3_)_2_CO	C_6_H_14_	Trimethyl Amine and Methyl Mercaptan
**TGS 2612**	S1	√	√	√	√								
**TGS 821**	S2	√	√			√		√					
**TGS 822**	S3	√	√		√			√	√		√	√	
**TGS 813**	S4	√	√	√	√	√		√					
**TGS 2602**	S5		√			√	√			√			
**TGS 2603**	S6		√			√	√						√
**TGS 2620**	S7	√	√		√	√		√					
**TGS 2610**	S8	√	√	√	√	√							

**Table 2:** Training and testing time taken by the algorithms to train and test with the data samples of banana, sapodilla, and pineapple, each at three ripeness states.

Algorithm	Big *O* Complexity	Train Time (s)	Test Time (s)
*k*-NN	*O*(*LM*(*N* + *k*))	0.2175	0.2175
SVM	*O*(*NL*^2^*M*^2^) to *O*(*NL*^3^*M*^3^)	0.7452	0.0461
GRNN	*O*(*I*_GRNN_)	0.3922	0.0946
RBFNN	*O*(*I*_RBFNN_) to *O*(*I^2^*_RBFNN_)	0.9922	0.0230
MLPNN	*O*(*I*^2^_MLPNN_)	0.8652	0.0153
MMM	*O*(*M*) to *O*(*M* + *I_ties_*(*N* + *k*))	0.1874	0.0047

**Table 3:** Misclassification error and correct classification rate of the classification algorithms during testing with validation and test data set from trained classes, i.e., banana, sapodilla, and pineapple each at three ripeness states, with nine classes in total. Results are averaged over 10 simulations. In the table *k*-NN, SVM, GRNN, RBFNN, MLPNN, and MMM are abbreaviated forms of *k*-nearest neighbor, support vector machine, generalized regression neural network, radial basis function neural network, multilayer perceptron neural network, and minimum-maximum-mean, respectively.

Algorithm	False Negative (Misclassification Error)		True Positive (Correct Classification)	
	Number of Test Samples Misclassified/Total Number of Test Samples	Percent	Number of Test Samples Correctly Classified/Total Number of Test Samples	Percent
*k*-NN	1/54 ^1^	1.8519	53/54	98.1481
SVM	1/54	1.8519	53/54	98.1481
GRNN	0.5/27 ^1^	1.8519	26.5/27	98.1481
RBFNN	6.5/27	24.0740	20.5/27	75.9260
MLPNN	8/27	29.6296	19/27	70.3704
MMM	0.5/27	1.8519	26.5/27	98.1481

^1^ 54 and 27 are the sizes of test dataset for different algorithms performance test.

**Table 4:** R-square values of different algorithms for training, validation, and test dataset. In the table *k*-NN, SVM, MLPNN, RBFNN, GRNN, and MMM are abbreaviated forms of *k*-nearest neighbor, support vector machine, multilayer perceptron neural network, radial basis function neural network, generalized regression neural network, and minimum-maximum-mean, respectively.

Dataset	*k*-NN	SVM	MLPNN	RBFNN	GRNN	MMM
Training	0.9827	0.9889	0.9349	0.9503	0.9889	0.9867
Validation	0.9801	0.9872	0.9217	0.9314	0.9805	0.9872
Test	0.9819	0.9825	0.9258	0.9361	0.9815	0.9803

**Table 5:** Classification performance of the algorithms with 60 irrelevant data samples (i.e., data from mango ripening stages). In the table *k*-NN, SVM, GRNN, RBFNN, MLPNN, and MMM are abbreaviated forms of *k*-nearest neighbor, support vector machine, generalized regression neural network, radial basis function neural network, multilayer perceptron neural network, and minimum-maximum-mean, respectively.

Classification Method	Misclassification Of Irrelevant Data				True Negative (Irrelevant Data Classified to No Trained or Irrelevant Classes i.e., Correctly Rejected)	
	False Positive (Irrelevant Data Classified to Trained Classes)		Irrelevant Data Classified to Unknown Classes			
	Number of Samples	Percent	Number of Samples	Percent	Number of Samples	Percent
*k*-NN	60	100	0	0	0	0
SVM	60	100	0	0	0	0
GRNN	0	0	0	0	60	100
RBFNN	9	15	51	85	0	0
MLPNN	21	35	39	65	0	0
MMM	0	0	0	0	60	100
